# Basophils, high-affinity IgE receptors, and CCL2 in human anaphylaxis

**DOI:** 10.1016/j.jaci.2016.12.989

**Published:** 2017-09

**Authors:** Peter Korosec, Paul J. Turner, Mira Silar, Peter Kopac, Mitja Kosnik, Bernhard F. Gibbs, Mohamed H. Shamji, Adnan Custovic, Matija Rijavec

**Affiliations:** a University Hospital of Respiratory and Allergic Diseases, Golnik, Slovenia; b Section of Paediatrics and MRC and Asthma UK Centre in Allergic Mechanisms of Asthma, Imperial College London, London, United Kingdom; c Medway School of Pharmacy, University of Kent, Chatham, United Kingdom; d Section of Allergy and Clinical Immunology, National Heart and Lung Institute and MRC and Asthma UK Centre in Allergic Mechanisms of Asthma, Imperial College London, London, United Kingdom

**Keywords:** Anaphylaxis, basophils, CD63 activation, FcεRI expression, CCL2, serum tryptase, CPA3, Carboxypeptidase A3, CRTH2, Chemoattractant receptor–homologous molecule expressed on T_H_2 lymphocytes, DBPCFC, Double-blind, placebo-controlled food challenge, ED, Emergency department, HDC, L-histidine decarboxylase, PMN, Polymorphonuclear leukocyte, ROC, Receiver operating curve, TSLP, Thymic stromal lymphopoietin

## Abstract

**Background:**

The role of basophils in anaphylaxis is unclear.

**Objective:**

We sought to investigate whether basophils have an important role in human anaphylaxis.

**Methods:**

In an emergency department study we recruited 31 patients with acute anaphylaxis, predominantly to Hymenoptera venom. We measured expression of basophil activation markers (CD63 and CD203c); the absolute number of circulating basophils; whole-blood *FCER1A*, carboxypeptidase A3 *(CPA3)*, and L-histidine decarboxylase *(HDC)* gene expression; and serum markers (CCL2, CCL5, CCL11, IL-3, and thymic stromal lymphopoietin) at 3 time points (ie, during the anaphylactic episode and in convalescent samples 7 and 30 days later). We recruited 134 patients with Hymenoptera allergy and 76 healthy control subjects for comparison. We then investigated whether the changes observed during venom-related anaphylaxis also occur during allergic reactions to food in 22 patients with peanut allergy undergoing double-blind, placebo-controlled food challenge to peanut.

**Results:**

The number of circulating basophils was significantly lower during anaphylaxis (median, 3.5 cells/μL) than 7 and 30 days later (17.5 and 24.7 cells/μL, *P* < .0001) and compared with those in patients with venom allergy and healthy control subjects (21 and 23.4 cells/μL, *P* < .0001). *FCER1A* expression during anaphylaxis was also significantly lower than in convalescent samples (*P* ≤ .002) and control subjects with venom allergy (*P* < .0001). CCL2 levels (but not those of other serum markers) were significantly higher during anaphylaxis (median, 658 pg/mL) than in convalescent samples (314 and 311 pg/mL at 7 and 30 days, *P* < .001). Peanut-induced allergic reactions resulted in a significant decrease in circulating basophil counts compared with those in prechallenge samples (*P* = .016), a decrease in *FCER1A* expression (*P* = .007), and an increase in CCL2 levels (*P* = .003).

**Conclusions:**

Our findings imply an important and specific role for basophils in the pathophysiology of human anaphylaxis.

Anaphylaxis is a potentially life-threatening, rapidly progressing systemic allergic reaction that can lead to death caused by airway obstruction or vascular collapse after exposure to allergens, including insect venom, foods, and medication.^1^ Mast cell activation is postulated to have a pivotal role in anaphylaxis,^2^ and an increase in serum mast cell tryptase levels can confirm the diagnosis.^1^ However, in subjects experiencing anaphylaxis, it is not unusual to find normal serum tryptase levels in the context of increased plasma histamine levels,^3^, ^4^, ^5^ suggesting that anaphylaxis might also involve basophil activation. However, there are few published data demonstrating a direct contribution of basophils to IgE-mediated anaphylaxis in human subjects.

Mast cells enter tissues as immature progenitors, where they undergo the final stages of their development and remain resident *in situ* for weeks or months. In contrast, basophils typically mature in hematopoietic tissues and subsequently circulate in the blood, with a half-life of less than 1 week.^6^ Local allergen challenge studies in human subjects have demonstrated an influx of basophils to inflammatory sites within several hours of allergen exposure, demonstrating the existence of mechanisms for basophil recruitment from the circulation to the site of allergen exposure.^7^, ^8^, ^9^ Both mast cells and basophils can rapidly secrete histamine and similar (but not necessarily identical) mediators and cytokines after IgE cross-linking.^2^ In murine studies basophils contribute to IgG-mediated anaphylaxis.^10^ In contrast, human basophils cannot be activated through IgG receptors, and their function is inhibited by IgG-mediated triggering through FcγRIIb receptors; moreover, they lack protease-activated receptors and antigen-presenting functions.^11^, ^12^

We hypothesized that basophils play an important role in human anaphylaxis and specifically that (1) basophils are activated during human anaphylaxis, (2) there is a basophil migration during anaphylaxis, and (3) basophil-related biomarkers might be useful to confirm anaphylaxis. We addressed our hypotheses in a series of interlinked studies. First, in an emergency department (ED) study we investigated the upregulation of CD63 expression (the most commonly used basophil activation marker^13^) during and after anaphylaxis (predominantly caused by Hymenoptera venom allergy). We monitored the absolute numbers of circulating basophils; the corresponding whole-blood gene expression of *FCER1A*, carboxypeptidase A3 *(CPA3)*, and L-histidine decarboxylase *(HDC)*; and serum levels of the major basophil chemotactic factors, including the CCR2 ligand CCL2 and the CCR3 ligands CCL11 and CCL5.^14^, ^15^ We also measured levels of T cell–derived IL-3 (an important basophil priming and growth factor) and epithelial cell–derived thymic stromal lymphopoietin (TSLP), which promotes IL-3–independent basophil development and activation.^6^, ^16^, ^17^ We then proceeded to assess whether the changes seen during venom-related anaphylaxis also occur during allergic reactions to food under the controlled setting of an oral double-blind, placebo-controlled food challenge (DBPCFC) in patients with peanut allergy.

## Methods

### Study participants

#### ED study

We prospectively recruited 31 patients (13 female patients; age, 18-79 years) presenting with an acute episode of anaphylaxis to the ED of University Hospital Golnik, Slovenia (June-August 2011 and July-November 2013). Reaction severity was graded according to the Mueller criteria.^18^ We collected blood samples during the reaction (at presentation to the ED) and in convalescent samples 7 and/or 30 days after the anaphylactic episode (see Table E1 in this article's Online Repository at www.jacionline.org).

#### Hymenoptera control subjects with venom allergy and healthy subjects

We recruited 2 groups of control participants for comparisons: (1) 134 patients (49 female patients; age, 23-67 years) with confirmed venom anaphylaxis from whom-blood samples were obtained at least 2 months after the last sting reaction and before initiation of venom immunotherapy and (2) 76 healthy control subjects (47 female subjects; age, 17-79 years).

Seventeen healthy subjects received a single dose of 64 mg of oral methylprednisolone and were monitored for up to 24 hours after the treatment to assess for possible confounding by treatment with corticosteroids and its effect on basophil activation, absolute cell count, *FCER1A* expression, and soluble markers (see Table E2 in this article's Online Repository at www.jacionline.org).

#### Peanut allergy study

We recruited 22 patients with peanut allergy (see Table E3 in this article's Online Repository at www.jacionline.org) in whom allergy was confirmed by using DBPCFCs (details are shown in the Methods section in this article's Online Repository at www.jacionline.org). Blood samples were collected before challenge, at cessation of challenge because of the onset of objective symptoms (but before administration of any treatment),^19^ and 2 to 4 hours after challenge.

Ethical approval was obtained from the Slovenian National Medical Ethics Committee (ED study and control participants) and the London Central Research Ethics Committee (peanut allergy study). All subjects provided written informed consent.

### Basophil activation, absolute cell count, gene expression, and serum markers

Detailed methodology is described in the Methods section in this article's Online Repository. Briefly, expression of CD63 and CD203c (markers of basophil activation) and enumeration of basophils (CD123^+^HLA-DR^−^ cells), lymphocytes, and polymorphonuclear leukocytes (PMNs) were determined by means of flow cytometry, as previously described.^20^, ^21^, ^22^ In samples from patients with peanut allergy, we determined the absolute basophil count using a similar methodology, with basophils identified as chemoattractant receptor–homologous molecule expressed on T_H_2 lymphocytes (CRTH2)–positive CD303^−^CD123^+^ cells.^23^

*FCER1A*, *CPA3*, and *HDC* gene expression was analyzed in whole-blood samples (PAXgene; PreAnalytiX, Hombrechtikon, Switzerland), as previously described.^22^

We measured serum concentrations of CCL2, CCL5, CCL11, IL-3, and TSLP by using ELISA, according to the manufacturers' instructions (Quantikine; R&D Systems, Minneapolis, Minn and Abcam, Cambridge, United Kingdom). For IL-3 measurements, we also performed spiking experiments (see the Methods section in this article's Online Repository). We measured serum total tryptase (α+β) levels with the ImmunoCAP 100 (Thermo Fisher, Uppsala, Sweden); tryptase concentrations that exceeded 11.4 μg/L were considered increased.

### Statistical analysis

The distribution of data was assessed by using the D'Agostino and Pearson test. We used appropriate nonparametic and parametric tests for comparisons between groups, including the Wilcoxon signed-rank test, Mann-Whitney *U* test, *t* test with the Welch correction, and Pearson correlation. Data are expressed as medians unless otherwise stated. We compared the performance of basophil-related biomarkers in discriminating between patients with and without anaphylactic reactions using receiver operating characteristic (ROC) curve analysis. Analyses were performed with GraphPad Prism software (GraphPad Software, La Jolla, Calif).

## Results

### Study participants

#### ED study and control subjects

**Fig E1:**
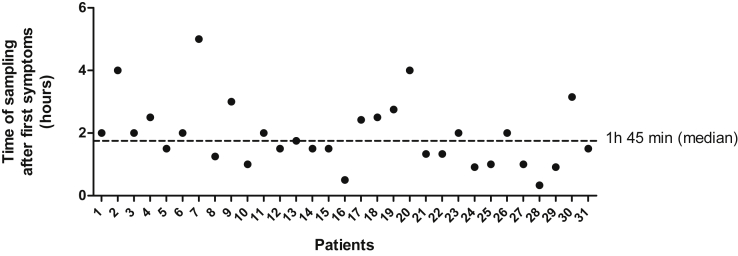
Time between symptom onset and blood sample collection in ED patients with acute anaphylactic reactions.

Fig E1 and Table E1 in this article's Online Repository at www.jacionline.org show detailed information on demographic characteristics, clinical and emergency treatment, and sampling data of 31 ED patients. The reaction was caused by an insect sting in 28 patients. The median time from symptom onset to sample collection was 105 minutes (range, 20 minutes to 5 hours; see Fig E1). Convalescent samples were collected from 28 patients 7 days after the anaphylactic episode and from 23 patients after 30 days (see Table E1); 2 patients provided samples 24 hours after the acute episode.

We measured basophil activation and counts in all ED patients and control subjects and serum tryptase levels in all ED patients and control subjects with venom allergy (see Table E4 in this article's Online Repository at www.jacionline.org). We ascertained gene expression in 15, chemokine and IL-3 levels in 17, and TSLP levels in 14 ED patients and analyzed *FCER1A* expression in 37 control subjects with venom allergy and CCL2 levels in 71 healthy control subjects (see Table E4).

#### Peanut allergy study

Basophil counts were determined in 22 patients with peanut allergy before and during both the active and placebo arms of the DBPCFC. CCL2 levels (n = 22) and *FCER1A* expression (n = 12) were ascertained during the active arm of the DBPCFC.

### Basophil markers in ED study and control subjects

#### Basophil activation

**Fig E2:**
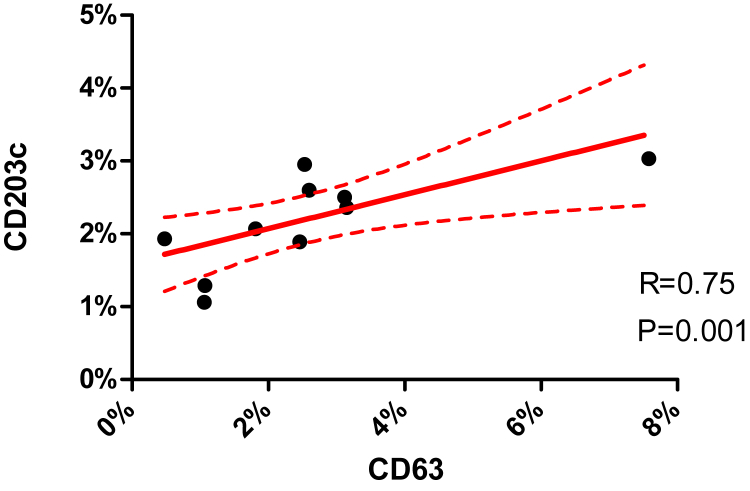
Correlation between basophil CD63 and CD203c activation in ED patients with acute anaphylactic reactions.

The percentage of CD63-activated basophils in ED patients during anaphylactic episodes was low (median, 3.8%). These values were marginally higher compared with those 7 (median, 2.9%; *P* = .01) and 30 (median, 2.9%; *P* = .05; Fig 1, *A*) days later. Only 4 patients had greater than 5% activated basophils, and only 1 exhibited activation of greater than 10%. This was mirrored by a small but significantly higher percentage of CD63-activated basophils during anaphylaxis compared with that seen in control subjects with venom allergy (median, 3.1%; *P* = .01) or healthy control subjects (median, 2.4%; *P* = .001; Fig 2, *A*). Expression of the activation marker CD203c correlated highly with that of CD63 (see Fig E2 in this article's Online Repository at www.jacionline.org).

**Fig 1:**
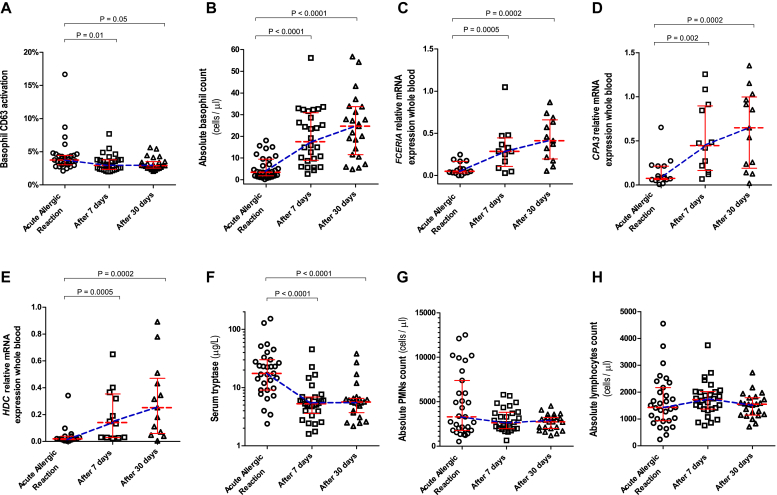
Basophil CD63 activation **(A)**; absolute basophil counts **(B)**; whole-blood *FCER1A***(C)**, *CPA3***(D)** and *HDC***(E)** expression; serum tryptase levels **(F)**; and PMN **(G)** and lymphocyte **(H)** absolute counts in ED patients during acute anaphylactic reactions to Hymenoptera venom and 7 and 30 days after the anaphylactic episode. *Horizontal lines* represent median values with interquartile ranges.

**Fig 2:**
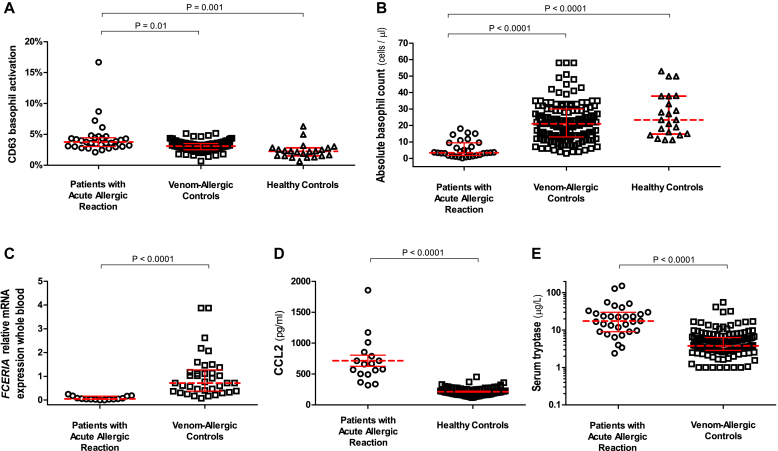
Comparison of basophil CD63 activation **(A)**, absolute basophil counts **(B)**, whole-blood *FCER1A* gene expression **(C)**, CCL2 serum concentrations **(D)**, and serum tryptase levels **(E)** between patients with acute anaphylactic reactions to Hymenoptera venom on ED presentation and patients with venom allergy or healthy control subjects. *Horizontal lines* represent median values with interquartile ranges.

#### Circulating basophils

The absolute number of circulating basophils was significantly lower during reactions (median, 3.5 cells/μL) compared with those 7 and 30 days later (17.5 and 24.7 cells/μL, respectively; *P* < .0001; Fig 1, *B*). This marked decrease (median, 83%; range, 53% to 99%) was evident in 30 of 31 patients. Basophil numbers in ED patients during the acute reaction were significantly lower compared with those in control subjects with venom allergy and healthy subjects (median, 21 and 23.4 cells/μL, respectively; *P* < .0001; Fig 2, *B*).

#### Gene expression

**Fig 3:**
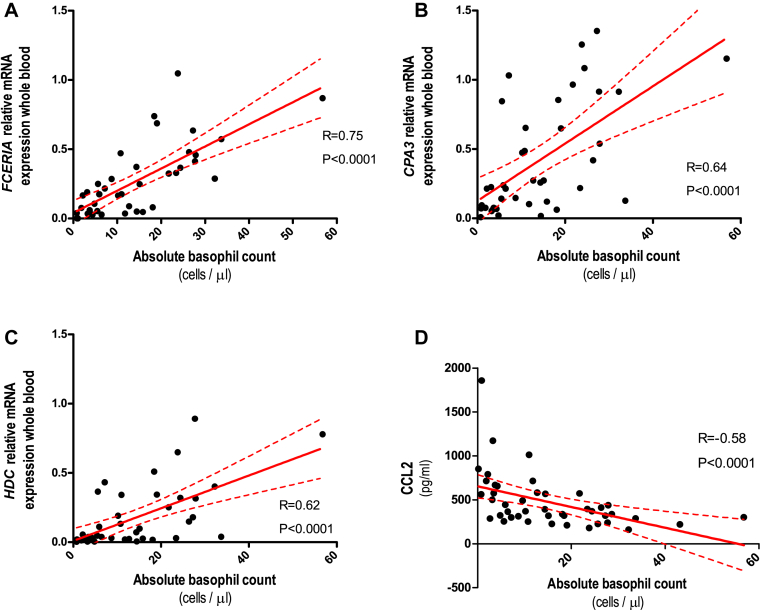
Correlation between absolute basophil counts and whole-blood *FCER1A***(A)**, *CPA3***(B)**, and *HDC***(C)** gene expression and serum CCL2 concentrations **(D)** in patients with acute anaphylactic reactions presenting to the ED.

**Fig E3:**
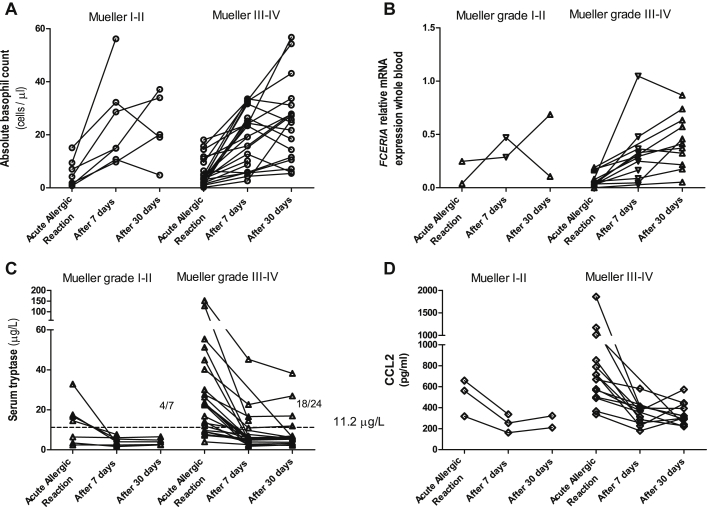
Absolute basophil counts **(A)**, whole-blood *FCER1A* gene expression **(B)**, serum tryptase levels **(C)**, and CCL2 serum concentrations **(D)** in ED patients divided according to the severity of acute allergic reactions (Mueller grade I-II vs grade III-IV) and then 7 and 30 days after the episode. The threshold for diagnostically positive tryptase measurement was set at 11.2 μg/L. Data are presented as a person-to-person scatter plot.

We observed significantly lower expression of *FCER1A*, *CPA3*, and *HDC* during the acute reaction compared with expression 7 and 30 days later (*P* ≤ .002; Fig 1, *C-E*; median decrease, 89% [range, 54% to 100%], 80% [range, 29% and 98%], and 86% [range, 57% to 98%] for *FCER1A*, *CPA3*, and *HDC*, respectively). *FCER1A* expression in ED patients during reactions was significantly lower compared with that in control subjects with venom allergy (*P* < .0001; Fig 2, *C*). Gene expression correlated highly with the absolute number of circulating basophils (*r* = 0.75, *r* = 0.64, and *r* = 0.62 [*P* < .0001] for *FCER1A*, *CPA3*, and *HDC*, respectively; Fig 3, *A-C*). Of note, we observed lower basophil counts and *FCER1A* expression in ED patients across different reaction severities (Mueller grade I-II and III-IV; see Fig E3, *A* and *B*, in this article's Online Repository at www.jacionline.org).

#### Serum markers

**Fig 4:**
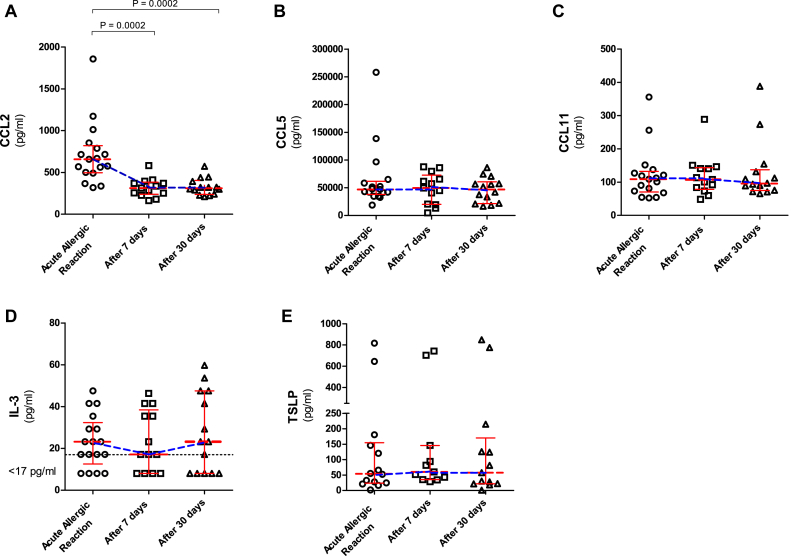
Serum CCL2 **(A)**, CCL5 **(B)**, CCL11 **(C)**, IL-3 **(D)**, and TSLP **(E)** levels in ED patients during acute anaphylactic reactions to Hymenoptera venom and 7 and 30 days after the anaphylactic episode. *Horizontal lines* represent median values with interquartile ranges.

CCL2 concentrations in ED patients during reactions (median, 658 pg/mL) were significantly higher than those measured in convalescent samples taken 7 and 30 days later (median, 314 and 311 pg/mL, respectively; *P* = .0002; Fig 4, *A*) and compared with 71 healthy control subjects (median, 201 pg/mL; *P* < .0001; Fig 2, *D*). CCL2 concentrations increased during the acute reaction (median increase, 113%; range, 50% to 477%) in all 17 patients (Mueller grade I-II and III-IV; see Fig E3, *D*). There was a significant negative correlation between serum CCL2 levels and the absolute number of circulating basophils (*r* = −0.58, *P* < .0001; Fig 3, *D*). There were no differences between the 3 time points in CCL5 (46.9, 49.5, and 46.7 ng/mL), CCL11 (109, 108, and 96 pg/mL), IL-3 (23, 17, and 23 pg/mL), and TSLP (54, 60, and 58 pg/mL) levels (see Fig 4, *B-E*).

The median serum tryptase level in ED patients was significantly higher during the acute reaction (17.5 μg/L) than 7 and 30 days later (5.2 and 5.6 μg/L, respectively; *P* < .0001; Fig 1, *F*) and compared with that in control subjects with venom allergy (3.8 μg/L, *P* < .0001; Fig 2, *E*). By using a binary cutoff of 11.4 μg/L, tryptase levels were increased during the acute episode in 22 (71%) of 31 patients (4/7 with Mueller I-II and 18/24 with Mueller grade III-IV reactions; see Fig E3, *C*).

#### Other blood cells

There were no differences in PMN and lymphocyte absolute counts during acute reactions compared with those 7 and 30 days later (PMNs: median, 3292, 2618, and 2738 cells/μL, respectively [Fig 1, *G*]; lymphocytes: 1431, 1724, and 1547 cells/μL [Fig 1, *H*]). Of note, in some patients an increase in PMN counts to greater than 10,000 cells/μL and a decrease in lymphocyte counts to less than 500 cells/μL were observed (Fig 1, *G* and *H*).

#### Interassay variability and potential confounding by treatment

**Fig E4:**
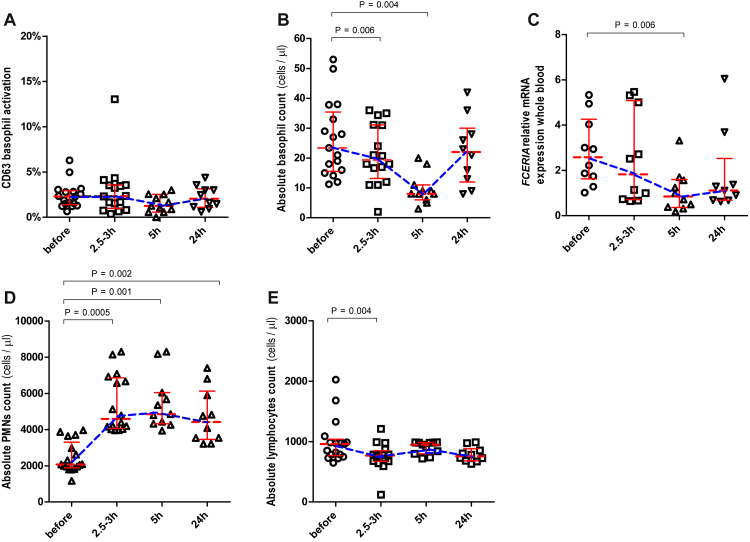
Basophil CD63 activation **(A)**, basophil absolute count **(B)**, whole-blood *FCER1A* gene expression **(C)**, and lymphocyte **(D)**, and PMN **(E)** absolute counts in healthy control subjects 2.5 to 3, 5, and 24 hours after the single dose of oral methylprednisolone (64 mg). *Horizontal lines* represent median values with interquartile ranges.

**Fig E5:**
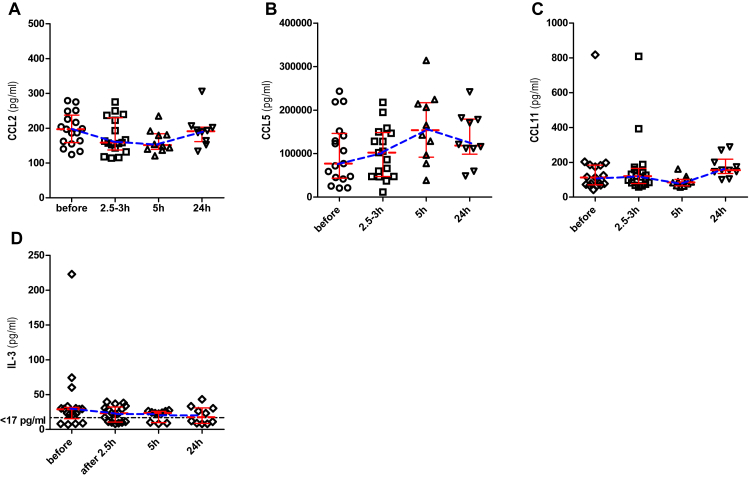
Serum concentrations of CCL2 **(A)**, CCL5 **(B)**, CCL11 **(C)**, and IL-3 **(D)** in healthy control subjects 2.5 to 3, 5, and 24 hours after the single dose of oral methylprednisolone (64 mg). *Horizontal lines* represent median values with interquartile ranges.

**Fig E6:**
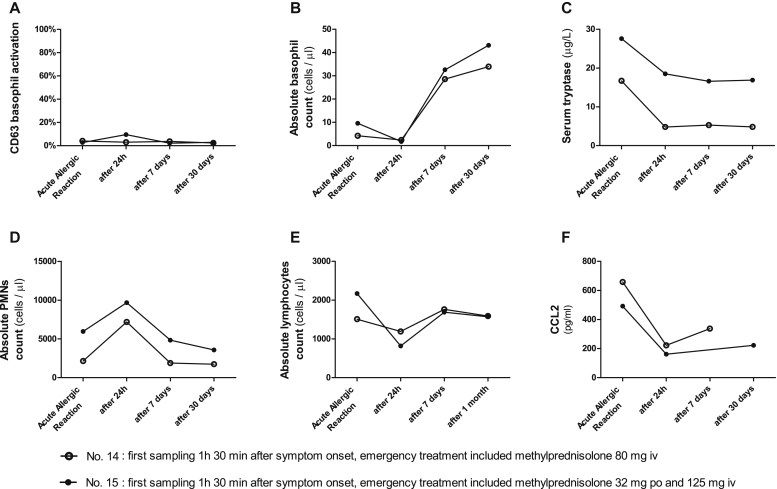
Basophil CD63 activation, absolute basophil counts, serum tryptase levels, PMN and lymphocyte absolute counts, and CCL2 serum concentrations in 2 ED patients (nos. 14 and 15, Table E1) sampled 1.5 hours, 24 hours, 7 days, and 1 month after the onset of symptoms. Both patients were treated with methylprednisolone. Data are presented as a before/after scatter plots.

**Fig E7:**
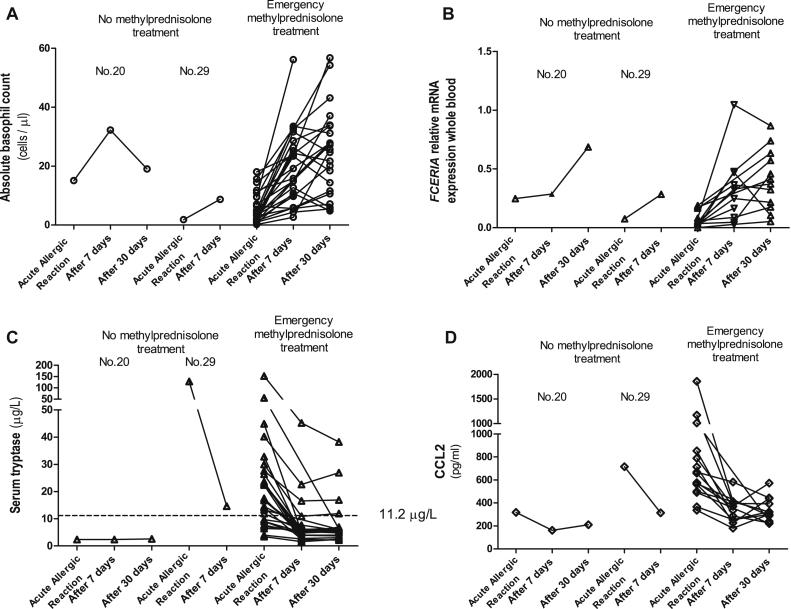
Basophil absolute counts, whole-blood *FCER1A* gene expression, serum tryptase levels, and CCL2 serum concentrations during acute anaphylactic reactions to Hymenoptera venom and 7 and 30 days after anaphylactic episodes in ED patients divided according to methylprednisolone treatment (patients 20 and 29 were not treated with methylprednisolone, Table E1). Data are presented as a person-to-person scatter plot.

Detailed results of these experiments are presented in Fig E4, Fig E5, Fig E6, Fig E7 in this article's Online Repository at www.jacionline.org. Briefly, there was a fast and substantial (>2-fold) increase in the absolute number of PMNs 2.5 to 3 hours after administration of methylprednisolone and a slower decrease in the absolute number of blood basophils and *FCER1A* expression (see Fig E4, *B-D*). There were no changes in CD63 activation and CCL2, CCL5, CCL11, and IL-3 levels (see Figs E4, *A*, and E5).

### Changes in basophil markers during acute allergic reactions to peanut

#### Circulating basophils

**Fig 5:**
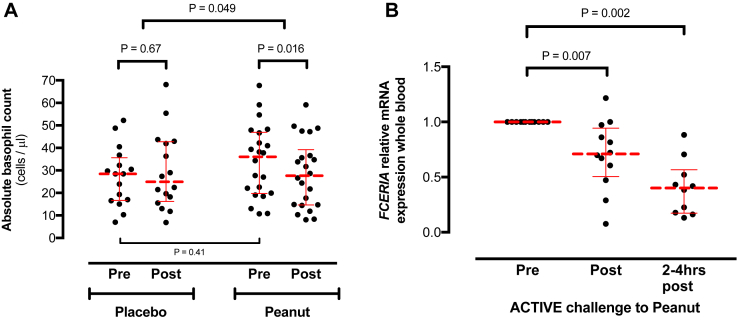
Absolute basophil counts **(A)** and whole-blood *FCER1A* gene expression **(B)** in patients with peanut allergy undergoing DBPCFCs to peanut. *Horizontal lines* represent median values with interquartile ranges.

There was a significant decrease in the absolute number of circulating basophils during the active arm of the DBPCFC compared with the matched prechallenge sample (*P* = .016); no such difference was observed during the placebo arm of the challenge (Fig 5, *A*). The decrease in circulating basophil counts was significantly greater in the active compared with placebo arms of the DBPCFC (median decrease, −23% [range, −57% to 33%] vs −4.5% [range, −36% to 141%], active vs placebo; *P* < .05).

#### *FCER1A* expression

During the active arm of the DBPCFC, there was a significant decrease from baseline in *FCER1A* expression both at the time of objective symptoms (but before administration of any treatment, *P* = .007) and 2 to 4 hours after the reaction (*P* = .002; Fig 5, *B*).

#### Serum CCL2 levels

**Fig 6:**
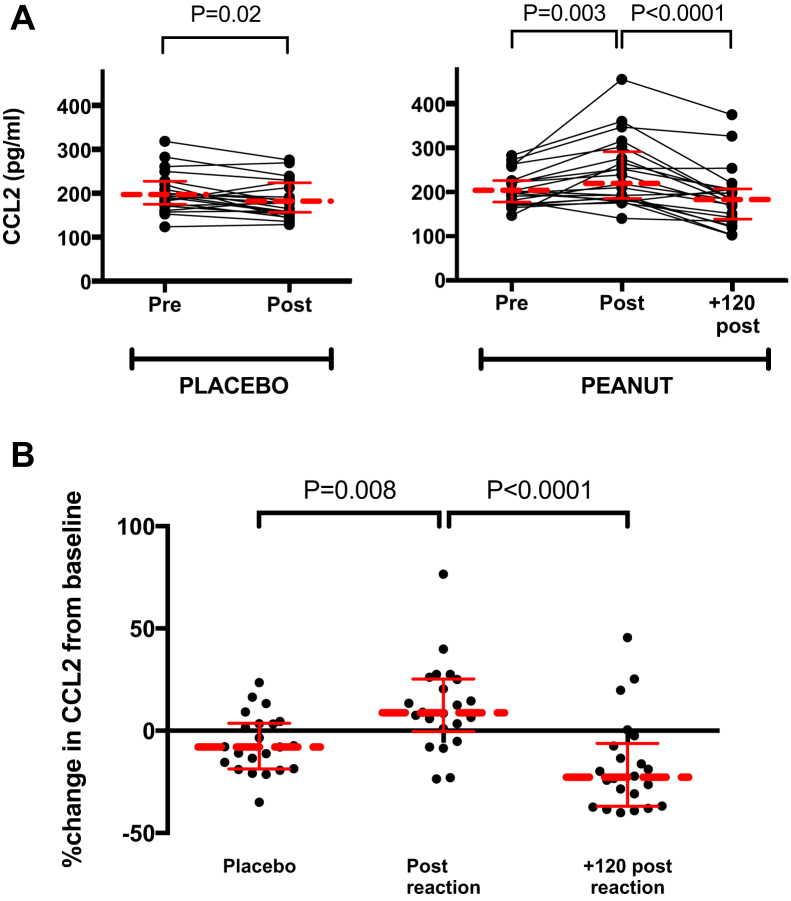
Serum CCL2 levels in allergic patients undergoing controlled DBPCFCs to peanut: **A,** absolute CCL2 levels; **B,** percentage change in CCL2 from baseline. *Horizontal lines* represent median values with interquartile ranges.

CCL2 levels increased significantly at the time of objective symptoms during the active arm of the DBPCFC compared with baseline levels (*P* = .003; Fig 6, *A*). CCL2 levels returned to baseline within 2 hours of symptom onset (Fig 6, *A* and *B*); the rate of increase in CCL2 levels was significantly greater in the active compared with placebo arms of the DBPCFC (*P* = .008; Fig 6, *B*).

### Predictors of anaphylaxis

**Fig E8:**
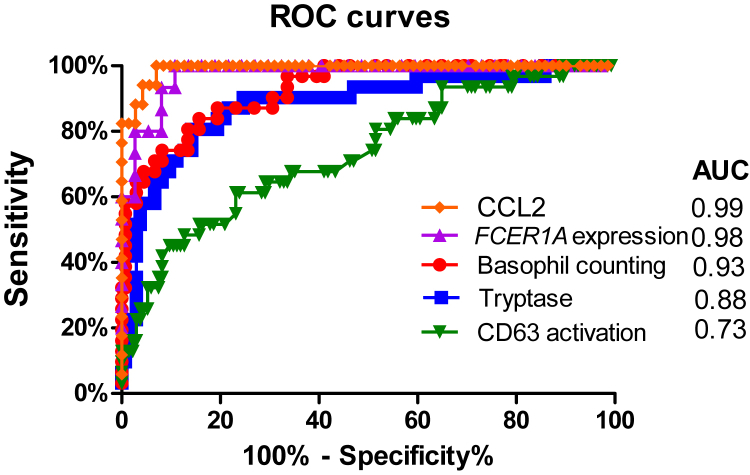
ROC curve analysis of basophil CD63 activation, absolute basophil counts, whole-blood *FCER1A* gene expression, CCL2 concentrations, and serum tryptase levels between patients with acute anaphylactic reactions to insect venoms on ED presentation and patients with venom allergy or healthy control subjects. *AUC*, Area under the curve.

As indicated by the estimated area under the ROC curve, CCL2 and *FCER1A* expressions were the most accurate readouts in discriminating between patients with anaphylactic reactions from those without, followed by basophil counts and tryptase levels: area under the ROC curve for CCL2, 0.99 (95% CI, 0.98-1); *FCER1A* expression, 0.98 (95% CI, 0.94-1); basophil count, 0.93 (95% CI, 0.88-0.97); tryptase level, 0.88 (95% CI, 0.81-0.95); and basophil activation, 0.73 (95% CI, 0.63-0.83; see Fig E8 in this article's Online Repository at www.jacionline.org (for further details, see the Results section in this article's Online Repository at www.jacionline.org). With a cutoff of greater than 334 pg/μL, the estimated sensitivity and specificity of CCL measurements were 94% and 96%, respectively, compared with 93% and 92% for *FCER1A* expression (cutoff, <0.2 cells/μL) and 87% and 81% for basophil counts (cutoff, >12 cells/μL).

## Discussion

Our study demonstrated a substantial (approximately 80%) reduction in circulating basophils during anaphylactic reactions to Hymenoptera venom. Decreased gene expression of *FCER1A*, *CPA3*, and *HDC* confirmed the flow cytometric data. We also observed an increase in CCL2 levels, which correlated with a decrease in circulating basophil counts. We replicated these findings in patients with peanut allergy experiencing allergic reactions during DBPCFCs to peanut. Compared with reactions in the ED, which were generally more severe, we observed more modest (but nonetheless significant) changes at the time of objective symptoms during peanut challenges. Taken together, these data suggest that anaphylaxis induces a rapid and considerable basophil migration. The mechanism of anaphylaxis-related basophil migration appears to be selective because no significant changes were seen for lymphocytes, PMNs, or chemotactic factors that might affect other effector cells, such as eosinophils (eg, CCL5 and CCL11).

#### Limitations

The nature of the management of anaphylaxis (including administration of high-dose corticosteroids) makes it difficult to exclude potential confounding by treatment and draw an unequivocal interpretation of the decrease in basophil counts in the ED setting. In our ED study 94% of patients received methylprednisolone, and 42% received epinephrine. Corticosteroids have a well-described effect on blood leukocytes, including an increase in circulating neutrophil counts and decrease in lymphocyte and basophil counts.^24^, ^25^ The kinetics of the response of various leukocytes to corticosteroid administration varies, with neutrophilia and lymphopenia preceding the onset of basopenia,^25^ which was confirmed in our study. Compared with healthy control subjects who received oral corticosteroids, the reduction in blood basophil (but not lymphocyte or PMN) counts was much greater and occurred at an earlier time in patients with acute anaphylaxis, suggesting that the changes in basophil counts were not related to treatment. Moreover, we replicated the observed changes in basophil markers in the controlled setting of patients with peanut allergy undergoing DBPCFCs where the study design allowed for blood sampling both before challenge and before any treatment. This avoids the issue of confounding by treatment (both with corticosteroids and epinephrine) and allows comparison with prereaction samples (something not possible in the ED setting). We acknowledge that 2 previous reports did not detect a change in absolute basophil counts after food challenge.^26^, ^27^ However, these studies involved fewer patients experiencing only mild allergic symptoms and used methods for basophil detection that were less sensitive and specific than those used in our study.

Several cytokines and chemokines are involved in basophil migration, with the CCR2 ligand CCL2 and the CCR3 ligand CCL11 eliciting the most potent migratory responses.^15^ However, there is a difference in the cellular specificity of these chemokines. CCR2 is virtually undetectable on human eosinophils,^28^ and thus CCL2 does not induce eosinophil migration, which is not the case for the CCR3 ligands CCL5 and CCL11.^29^ Therefore CCL2-mediated migration might represent a unique mechanism for the selective migration of human basophils in allergic reactions. However, in the present study we could not determine the cellular sources of CCL2 during acute reactions.

We could not answer the question of whether anaphylaxis is associated with extensive activation and degranulation of circulating basophils. Patients with anaphylaxis present to the ED up to hours after symptom onset, and it takes additional time to obtain informed consent and perform venipuncture. In our study the median time between symptom onset and sample collection was 105 minutes, which is comparable with previous ED studies.^4^, ^30^, ^31^ Plasma histamine levels, which correlate with anaphylactic symptoms,^32^, ^33^ typically peak within 5 to 10 minutes after the onset of anaphylaxis and subsequently decrease to baseline levels within 1 hour as a result of rapid catabolism. Consequently, the relatively modest increase in CD63 expression on basophils (a marker of basophil degranulation) might represent an underestimate of the peak basophil activation during acute reactions. In a recent open food challenge study of delayed responses to meat in patients sensitized to galactose-α-1,3-galactose, expression of CD63 was reported for more than 15% of basophils in 9 of 12 patients at symptom onset.^34^ This is consistent with our data, which also support more extensive basophil activation (typically up to 20% of basophils expressing CD63 and CD203c) during peanut-induced allergic reactions.^35^ In our ED study only 1 of 31 patients predominantly allergic to venom had greater than 15% CD63-activated basophils, despite the fact that the majority (24/31) experienced anaphylactic reactions of Mueller grade III or IV severity (with bronchospasm, airway obstruction, hypoxemia or hypotension, and collapse). Whether this difference is due to the unavoidable delay in sampling after symptom onset in the ED compared with the challenge setting or a difference in the extent of basophil activation for venom- versus food-induced allergic reactions is unknown. It is most likely that we detected only those basophils that remained in the circulation after the acute reaction (approximately 20% of the normal level of basophils) and not the basophils that had migrated out of the circulation.

#### Interpretation

Recent reports have implicated a specific effector role for basophils in acute allergic responses.^21^, ^36^, ^37^, ^38^ Studies that used oral food or nasal allergen challenge responses in omalizumab-treated adults with peanut^37^ or cat^36^ allergies have suggested that acute reactions might be basophil rather than mast cell dependent. Decreases in the basophil allergen responses after venom immunotherapy reflect the induction of tolerance to sting challenges.^21^ A recent study in children with peanut allergy suggested that an *in vitro* basophil activation test at baseline might correlate with reaction severity at subsequent food challenge.^38^ However, these *in vitro* studies could not confirm whether basophil activation actually contributes to the acute allergic reactions or is a surrogate marker of mast cell or overall IgE responsiveness. Thus studies investigating human basophils during allergic reactions *in vivo* are required. However, such studies in a controlled challenge setting are difficult because of the general consensus that patients who might experience severe anaphylactic reactions should be excluded. Moreover, reaction severity at challenge is generally limited by the controlled nature of the challenge (where allergen exposure is stopped at onset of objective symptoms). Therefore we combined an ED-based study in patients with venom allergy, which focused on basophil migration and/or activation during more severe anaphylaxis, with a study of peanut-induced allergic reactions during DBPCFCs in which patients tended to experience less severe reactions. Data from this latter study in patients with peanut allergy corroborated the findings from the ED study.

One interesting question that remains unanswered is when and where basophil activation occurs. Anti-IgE, anti-FcεRI, or allergen stimulation of basophils also promotes their migration and adherence to endothelial cells.^39^, ^40^ However, these stimuli might enhance basophil adherence to the vascular endothelium and migration at concentrations lower than the threshold required for basophil degranulation and histamine release.^39^, ^40^ Therefore IgE-mediated basophil migration might be induced without basophil degranulation. This suggests that basophils can be activated after migration or partly in circulation and partly after migration or might even migrate without activation. The different clinical severities and end-organ patterns of anaphylaxis^1^, ^2^ and the finding that serum mast cell tryptase levels are often within normal limits^3^, ^4^ suggest that local rather than generalized mast cell and/or basophil degranulation might predominate in some subjects. Additional studies are required to confirm these speculations.

The short timeframe within which the reduction in circulating basophil count occurred, coupled with previous findings that basophils are the granulocytes most resistant to apoptosis,^41^ suggest that anaphylaxis induces a prompt basophil migration rather than elimination by means of apoptosis. We did not observe a change in serum IL-3 or TSLP levels. This suggests that it is unlikely that basophil migration during anaphylaxis is related to changes in basophil development or homeostasis, a process that is IL-3 elicited for basophils that operate in an IgE-dependent manner or TSLP elicited for basophils that operate in a non–IgE-dependent manner.^6^ Our results are consistent with those of a recent study that demonstrated no changes in CCL11 or IL-3 levels during anaphylaxis.^30^

Risk assessment of patients with anaphylaxis is hampered by limitations in laboratory tests to confirm the diagnosis and predict its severity.^42^, ^43^ Currently, the only readily available laboratory test to confirm the diagnosis of anaphylaxis is the measurement of total tryptase levels in serum/plasma.^1^, ^2^ However, even when blood sampling is optimally timed, tryptase levels are often within normal limits, particularly for food-induced reactions.^3^, ^4^ In our study of predominantly venom-induced reactions, a diagnostic increase in the total tryptase level was seen in 71% of patients with anaphylaxis, which is comparable with other reports.^30^ Although other mediators have been proposed as potential biomarkers,^30^, ^31^, ^44^, ^45^, ^46^ these have not exhibited sufficient diagnostic utility or technical reproducibility to be used routinely.^1^, ^2^ Our results indicate that CCL2, *FCER1A* expression, and basophil counts might be useful biomarkers of anaphylaxis. However, a substantially broader assessment is required to validate these methods and replicate the findings.

#### Conclusions

Our data suggest a substantial migration of circulating basophils during anaphylaxis, which correlates with a significant increase in serum concentrations of the major basophil chemotactic factor CCL2. These findings suggest an important and specific role for basophils in the pathophysiology of human anaphylaxis.

**Key messages**

• Human anaphylaxis involves a substantial reduction in numbers of circulating basophils, which inversely correlate with serum CCL2 levels, a major basophil chemotactic factor.
• This decrease was confirmed by reduced whole-blood *FCER1A*, *CPA3*, and *HDC* gene expression.
• These data imply an important and specific role for basophils in the pathophysiology of human anaphylaxis.

## Figures and Tables

**Table E1:** Demographic and clinical data of patients with acute anaphylactic reactions recruited from the hospital ED

No.	Sex	Age (y)	Culprit	Mueller grade	Emergency treatment	Time from onset of reaction to blood collection	Previous anaphylaxis or VIT
1	M	41	Honeybee	4	aH1 (2 mg IV), ST (80 mg IV)	2 h, 7 d, 30 d	No
2	F	39	Honeybee	4	Epi (0.5 mg IM), aH1 (10 mg PO, 2 mg IV), ST (64 mg PO, 250 mg IV)	4 h, 7 d	1 y honeybee VIT in 2005
3	M	63	*Vespula* species	4	Epi (1.5 mg IM), ST (32 mg PO, 80 mg IV)	2 h, 7 d, 30 d	5 y *Vespula* VIT finished in 1999
4	F	54	*Vespula* species	2	Epi (0.5 mg SC), aH1 (2 mg IV), ST (125 mg IV)	2 h and 30 min, 7 d, 30 d	No
5	F	54	*Vespula* species	3	aH1 (2 mg IV), ST (125 mg IV)	1 h and 30 min, 7 d, 30 d	No
6	M	49	*Vespula* species	2	aH1 (10 mg PO, 2 mg IV), ST (32 mg PO, 125 mg IV)	2 h, 7 d, 30 d	No
7	M	32	Unknown Hymenoptera	2	aH1 (2 mg IV), ST (250 mg IV)	5 h, 7 d	No
8	M	49	*Vespula* species	3	aH1 (20 mg PO, 2 mg IV), ST (64 mg PO, 300 mg IV)	1 h and 15 min, 7 d, 30 d	*Vespula* VIT from 2009
9	F	40	*Vespula* species	3	aH1 (2 mg IV), ST (250 mg IV)	3 h, 7 d, 30 d	2010 *Vespula* species, grade 1
10	M	74	Honeybee	4	Epi (0.1 mg IV), aH1 (2 mg IV), ST (125 mg IV)	1 h, 7 d, 30 d	No
11	M	51	European hornet	4	aH1 (2 mg IV), ST (165 mg IV)	2 h, 7 d	No
12	M	28	*Vespula* species	3	aH1 (4 mg IV), ST (64 mg PO, 40 mg IV)	1 h and 30 min, 7 d	No
13	M	18	Honeybee	1	aH1 (4 mg IV), ST (80 mg IV)	1 h and 45 min, 7 d	No
14	M	42	Unknown Hymenoptera	2	aH1 (2 mg IV), ST (80 mg IV)	1 h and 30 min, 24 h, 7 d, 30 d	No
15	F	61	Unknown Hymenoptera	3	aH1 (10 mg PO, 2 mg IV), ST (32 mg PO, 125 mg IV)	1 h and 30 min, 24 h, 7 d, 30 d	No
16	F	20	European hornet	4	aH1 (20 mg PO, 2 mg IV) ST (64 mg PO, 125 mg IV)	30 min, 7 d, 30 d	2012 *Vespula* species, grade 1
17	F	70	Unknown Hymenoptera	3	aH1 (2 mg IV), ST (500 mg IV)	2 h and 25 min, 30 d	No
18	M	71	*Vespula* species	3	aH1 (2 mg IV), ST (125 mg IV)	2 h and 30 min, 7 d, 30 d	No
19	M	57	European hornet	4	aH1 (2 mg IV), ST (40 mg IV)	2 h and 45 min, 30 d	No
20	F	33	European hornet	1	No drugs administered	4 h, 7 d, 30 d	*Vespula* species, multiple times as child, grade 3
21	M	50	*Vespula* species	4	Epi (0.3 mg IM), aH1 (20 mg PO, 2 mg IV), ST (64 mg PO, 125 mg IV), bronchodilator (fenoterol, 0.5 mg; ipratropium bromide, 0.2 mg)	1 h and 20 min, 7 d, 30 d	*Vespula* species, 4 times since 2002, grade 4
22	M	48	Honeybee	4	Epi (0.3-0.5 mg IM), aH1 (2 mg IV), ST (>40 mg IV)	1 h and 20 min, 7 d, 30 d	Honeybee; 2009, 2011; grade 2
23	M	47	*Vespula* species	3	aH1 (2 mg IV), ST (80 mg IV)	2 h, 7 d, 30 d	No
24	M	62	European hornet	4	Epi (0.5 mg IM), aH1 (2 mg IV), ST (125 mg IV)	55 min, 30 d	Since 2007, VIT *Vespula*; since 2009, VIT honeybee
25	F	56	Unknown	4	Epi (0.3-0.5 mg IM), aH1 (2 mg IV), ST (125 mg IV)	<1 h, 7 d, 30 d	Two previous anaphylaxis, unknown trigger, grade 4
26	F	56	*Vespula* species	4	Epi (2 × 0.5 mg IM), aH1 (2 mg IV), ST (125 mg IV)	2 h, 7 d, 30 d	2010, *Vespula* species, grade 1
27	M	79	European hornet	3	aH1 (2 mg IV), ST (80 mg IV)	1 h, 7 d, 30 d	No
28	F	66	IV analgesic	4	Epi (0.3 mg IM), aH1 (2 mg IV), ST (80 mg IV)	20 min, 7 d, 30 d	No
29	F	56	Honeybee VIT	4	Epi (0.3 mg IM), aH1 (2 mg IV)	55 min, 7 d	2012, unknown Hymenoptera, grade 4
30	F	55	Honeybee	3	Epi (0.3 mg IV), aH1 (20 mg PO, 2 mg IV), ST (64 mg PO, 500 mg IV)	3 h and 10 min, 7 d	Honeybee VIT started in 2008 but stopped the same year
31	M	68	European hornet	4	Epi (0.3 mg IM), aH1 (2 mg IV), ST (125 mg IV)	1 h and 30 min, 7 d	No

*aH1*, Loratadine (PO) and/or Clemastine (IV); *Epi*, epinephrine; *F*, female; *IM*, intramuscular; *IV*, intravenous; *M*, male; *PO*, by mouth; *SC*, subcutaneous; *ST*, methylprednisolone; *VIT*, venom immunotherapy.

**Table E2:** Demographic data and sampling of healthy subjects after a single dose of oral methylprednisolone

No.	Sex	Age (y)	Single-dose oral methylprednisolone	Time of blood collection
1	F	41	64 mg	Just before ST, after 3 h
2	M	29	64 mg	Just before ST, after 3 h
3	F	28	64 mg	Just before ST, after 3 h
4	M	42	64 mg	Just before ST, after 5 h
5	F	32	64 mg	Just before ST, after 3 h
6	F	44	64 mg	Just before ST, after 2.5 h
7	F	37	64 mg	Just before ST, after 2.5 h
8	F	24	64 mg	Just before ST, after 2.5, 5, and 24 h
9	M	28	64 mg	Just before ST, after 2.5, 5, and 24 h
10	M	30	64 mg	Just before ST, after 2.5, 5, and 24 h
11	F	24	64 mg	Just before ST, after 2.5, 5, and 24 h
12	F	24	64 mg	Just before ST, after 2.5, 5, and 24 h
13	F	35	64 mg	Just before ST, after 2.5, 5, and 24 h
14	F	39	64 mg	Just before ST, after 2.5, 5, and 24 h
15	F	35	64 mg	Just before ST, after 2.5, 5, and 24 h
16	M	30	64 mg	Just before ST, after 2.5, 5, and 24 h
17	F	28	64 mg	Just before ST, after 2.5, 5, and 24 h

*F*, Female; *M*, male; *ST*, 64 mg of oral methylprednisolone.

**Table E3:** Demographic and clinical data relating to patients with peanut allergy undergoing DBPCFCs to peanut

	Overall cohort	Epinephrine administered at DBPCFC∗
No.	22	5
Age (y), median (range)	14.8 (8-36)	21.5 (12-26)
Male (%)	64	40
SPT to peanut (mm), median (range)	9 (5-16)	11 (9-11)
sIgE to peanut (kU_A_/L), median (range)	18.1 (3.1->100)	27.6 (13.5-61.4)
sIgE to rAra h 2 (kUA/L), median (range)	12.2 (0.23->100)	13.1 (12.2-52.9)
Grade of reaction at DBPCFC		
Mueller I/II	16	0
Mueller III	6	5

*SPT*, Skin prick test; *sIgE*, specific IgE.

∗ Intramuscular epinephrine was administered for any lower respiratory and/or cardiovascular symptoms.

**Table E4:** Detailed information on the number of participants in whom we assessed basophil activation, absolute cell counts, gene expression, and soluble markers

	Basophil absolute count	Basophil activation (CD63)	Basophil activation (CD203c)	*FCER1A*	*CPA3*	*HDC*	CCL2	CCL5	CCL11	IL-3	TSLP	Serum tryptase	PMN and lymphocyte absolute count
ED patients (n = 31)	31	31	9	15	15	15	17	17	17	17	14	31	31
Control subjects with venom allergy (n = 134)	134	134	–	37	–	–	–	–	–	–	–	134	–
Healthy control subjects (n = 76)	22	22	–	–	–	–	71	–	–	–	–		–

–, Not done.

## Methods

### DBPCFCs to peanut

DBPCFCs were conducted according to international consensus criteria (PRACTALL).^E1^ In brief, subjects underwent DBPCPCs over 2 separate days at least 7 days apart. On each day, subjects received increasing doses every 30 minutes of peanut protein (or placebo) at the following doses: 3, 10, 30, 100, 300, 1000, and 3000 mg until stopping criteria were met (as per PRACTALL consensus).^E1^ Blood samples were collected from a venous cannula sited before challenge and immediately snap-frozen or transferred without delay for flow cytometry.

### Basophil activation and absolute cell count

A precise volume of whole heparinized blood (100 μL) was incubated with fluorescein isothiocyanate–conjugated anti-CD63 mAb, phycoerythrin-conjugated anti-CD123 mAb, and peridinin-chlorophyll-protein complex–conjugated anti–HLA-DR mAb (BD Biosciences, San Jose, Calif), and thereafter, the samples were lysed, washed, fixed, and analyzed within 2 hours on a FACSCalibur flow cytometer (BD Biosciences). In a proportion of samples, we also added allophycocyanin-conjugated CD203c (Miltenyi Biotec, Auburn, Calif) for an additional activation analysis. The basophils were identified as low side-scatter, CD123^+^, and HLA-DR^−^ cells. The quantitative percentage determination of activated basophils (CD63^+^) was measured in FL1. Fluorescein isothiocyanate mouse IgG_1_ isotype control (BD Biosciences) was also tested to evaluate unspecific staining.

For the absolute basophil count (CD123^+^HLA-DR− cells), 50 μL of AccuCount Fluorescent microbeads (7.7 μm, 51,011 particles per 50 μL; Spherotech, Lake Forest, Ill) was added to the fixed samples before flow cytometric analysis. Lymphocytes and PMNs were gated according to lysed whole-blood forward-scatter/side-scatter characteristics. Absolute numbers of basophils, lymphocytes, and PMNs per microliter of whole blood were calculated by using the following equation:

$$\left(\frac{\mathrm{Number}\;\mathrm{of}\;\mathrm{cells}}{\mathrm{Number}\;\mathrm{of}\;\mathrm{events}\;\mathrm{per}\;\mathrm{microbead}\;\mathrm{region}}\right)\times \left(\frac{\mathrm{Number}\;\mathrm{of}\;\mathrm{microbeads}\;\mathrm{used}\;\mathrm{in}\;\mathrm{test}}{\mathrm{Volume}\;\mathrm{of}\;\mathrm{the}\;\mathrm{whole}\;\mathrm{blood}\;\mathrm{sample}}\right)$$

In samples from donors with peanut allergy undergoing DBPCFCs, absolute basophil counts were determined by using a similar methodology with 50 μL of CountBright microbeads (7 μm, 0.45-0.55 × 10^5^ beads/50 μL; Thermo Fisher), with basophils identified as CRTH2^+^CD303^−^CD123^+^ cells.^E2^ In a selection of samples, basophil counts were determined by using both microbeads to confirm equivalency.

### Gene expression

We analyzed gene expression of the α-subunit of the high-affinity IgE receptor (*FCER1A*, Hs00175232_m1), *CPA3* (Hs00157019_m1), and *HDC* (Hs00157914_m1). FcεRI is expressed on mast cells and basophils as tetramers (αβγ_2_) and on antigen-presenting cells, although at substantially lower levels, as trimers (αγ_2_).^E3^ CPA3 is expressed in mast cells and basophils and can be expressed in populations of T-cell progenitors and thymic T cells and in some hematopoietic progenitor cells.^E4^ HDC catalyzes the formation of histamine from L-histidine, and in hematopoietic cell lineages the gene is expressed only in mast cells and basophils.^E5^

Total RNA was isolated from whole-blood samples by using the PAXgene Blood miRNA Kit (PreAnalytiX) and quantified with the Qubit fluorometer (Thermo Fisher Scientific, Waltham, Mass). After reverse transcription, cDNA was quantified by using real-time PCR (ABI PRISM 7500 Real-Time PCR System; Applied Biosystems, Foster City, Calif) at standard conditions with TaqMan Universal PCR Master Mix (Thermo Fisher Scientific). Expression levels were normalized against ribosomal 18s RNA Endogenous Control (Thermo Fisher Scientific). All measurements were performed in triplicate for each sample, and time point and relative expressions were analyzed by using the ΔΔ cycle threshold method.

### IL-3 spiking experiments

For IL-3 measurements, we performed spiking experiments with *Escherichia coli*–derived recombinant human IL-3 protein (from R&D Systems, Minneapolis, Minn) in which a known amount of recombinant protein was spiked into a sera sample with an undetectable intrinsic IL-3 concentration (ie, <17 pg/mL according to our detection limit) and run in the ELISA. We successfully recovered samples spiked with 250, 125, 62.5, or 32.5 pg/mL recombinant human IL-3 protein but not samples spiked with known concentrations of 15.6, 7.8, or 3.9 pg/mL recombinant human IL-3 protein. This sensitivity is within the range of the minimum detectable concentration of IL-3 (from 3.46-57.4 pg/mL) evaluated by using the commercial Human IL-13 Quantikine ELISA Kit (R&D Systems).

## Results

### Interassay coefficient of variation

We estimated an interassay coefficient of variation of 6.7% for the absolute basophil count and 4.8% for basophil CD63 activation using repeated measurements in 5 healthy control subjects.

### Effects of oral corticosteroids on basophil markers and other blood cells

We followed 17 healthy subjects for up to 24 hours after a single dose of 64 mg of oral methylprednisolone (Table E2).

#### Basophil activation

There was no significant effect of treatment with oral corticosteroids on basophil (CD63) activation (Fig E4, *A*).

#### Circulating basophils

We identified a small but statistically significant decrease in the absolute count of blood basophils (from a median of 23.4 to 19.7 cells/μL; median decrease, 19%; *P* = .006). However, a major decrease (to 8 cells/μL; median decrease, 67%; *P* = .004) was observed 5 hours after methylprednisolone administration (Fig E4, *B*). Basophil counts returned to normal after 24 hours (to 22 cells/μL).

#### Gene expression

We observed a small and nonsignificant decrease in *FCER1A* expression 2.5 to 3 hours after methylprednisolone intake, followed by a substantial decrease after 5 hours, which corresponded to a major decrease in basophil numbers (median decrease, 63%; *P* = .006; Fig E4, *C*). *FCER1A* expression did not differ between the baseline level and the level 24 hours after methylprednisolone.

#### Other blood cells

There was a significant increase in the absolute number of blood PMNs 2.5 to 3 hours after methylprednisolone intake (>2-fold increase; median, 2070-4585 cells/μL; *P* = .0005; Fig E4, *D*). This increase was also seen after 5 hours (4853 cells/μL, *P* = .001) and 24 hours (4422 cells/μL, *P* = .002; Fig E4, *D*).

After 2.5 to 3 hours, there was a small but statistically significant decrease in the number of blood lymphocytes (median, 960 to 768 cells/μL; *P* = .004; Fig 4, *E*). There was no difference in lymphocyte counts 5 and 24 hours after methylprednisolone compared with baseline values (Fig E4, *E*).

#### Serum markers

There was no significant effect of treatment with oral corticosteroids on CCL2, CCL5, CCL11, or IL-3 levels (Fig E5).

#### ED patients

In 2 ED patients (nos. 14 and 15, Table E1) in whom we collected samples during the acute anaphylactic episode and 24 hours later and who received emergency treatment with systemic corticosteroids, during the acute allergic reaction, we observed changes in basophil counts, CCL2 levels, and tryptase levels but not in PMN and lymphocyte counts (Fig E6). The increase in PMN counts and the decrease in lymphocyte counts became evident only at the 24-hour sampling point (Fig E6). The decrease in basophil counts and *FCER1A* expression, as well as the increase in tryptase and CCL2 levels, were also observed in 2 ED patients (nos. 20 and 29, Table E1) who did not receive treatment with corticosteroids (Fig E7).

### Predictors of anaphylactic reactions

We compared the performance of basophil counts, basophil activation, tryptase levels, and CCL2 and FcεRI expression in discriminating between patients with anaphylactic reactions and those without using an ROC curve analysis. For the control groups, we used patients with confirmed venom allergy from whom samples were obtained at least 2 months after the last sting reaction and before venom immunotherapy was initiated (134 control subjects for basophil counts, basophil activation, and tryptase levels and 37 control subjects for *FCER1A* expression) or healthy control subjects (54 control subjects for CCL2 levels).

When we compared values at the time of the reaction with those 1 month later, the estimated areas under the ROC curve were 0.92 (95% CI, 0.83-1), 0.93 (95% CI, 0.84-1), and 0.92 (95% CI, 0.86-0.99) for CCL2 levels, *FCER1A* expression, and basophil counts, respectively.
